# (2S*)-2-Ammonio-3-(1*H*-indol-3-yl)propionate pyridine-2,4-dicarboxylic acid ethanol solvate

**DOI:** 10.1107/S1600536810014017

**Published:** 2010-04-21

**Authors:** Kai Di

**Affiliations:** a College of Chemistry and Chemical Engineering, Qiqihar University, Qiqihar 161006, People’s Republic of China

## Abstract

In the title compound, C_11_H_12_N_2_O_2_·C_7_H_5_NO_4_·C_2_H_6_O, the (2S*)-2-amino-3-(1*H*-indol-3-yl)propionic acid is present in the zwitterionic form. In the crystal structure, 2-amino-3-(1*H*-indol-3-yl)propionic acid mol­ecules and pyridine-2,4-dicarb­oxylic acid mol­ecules are linked through strong inter­molecular O—H⋯O and N—H⋯O hydrogen bonds, forming layers parallel to (100). The layers are linked through the ethanol mol­ecules *via* somewhat weaker inter­molecular O—H⋯O and N—H⋯O hydrogen bonds, forming thus a three-dimensional network. Weak C—H⋯O and N—H⋯N hydrogen bonding and π–π inter­actions between the aromatic rings are also present.

## Related literature

For supra­molecular structures with imino, carboxyl­ate and pyridine groups inter­connected *via* inter­molecular hydrogen bonds, see: Broker & Tiekink (2010 ▶); Hemamalini & Fun (2010 ▶); Narimani & Yamin (2010 ▶); Pourayoubi *et al.* (2010 ▶). For a description of the Cambridge Structural Database, see: Allen (2002 ▶). For hydrogen bonding, see: Desiraju & Steiner (1999 ▶).
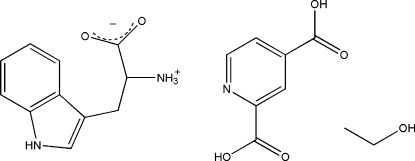


## Experimental

### 

#### Crystal data

C_11_H_12_N_2_O_2_·C_7_H_5_NO_4_·C_2_H_6_O
*M* *_r_* = 417.41
Triclinic, 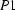

*a* = 7.0320 (14) Å
*b* = 7.7590 (16) Å
*c* = 9.5800 (19) Å
α = 85.44 (3)°
β = 81.89 (3)°
γ = 71.84 (3)°
*V* = 491.34 (19) Å^3^
*Z* = 1
Mo *K*α radiation
μ = 0.11 mm^−1^
*T* = 298 K
0.27 × 0.23 × 0.22 mm

#### Data collection

Bruker SMART 1000 CCD diffractometer
Absorption correction: multi-scan (*SADABS*; Sheldrick, 1996 ▶) *T*_min_ = 0.971, *T*_max_ = 0.977
4115 measured reflections
2092 independent reflections
1815 reflections with *I* > 2σ(*I*)
*R*_int_ = 0.024

#### Refinement

*R*[*F* ^2^ > 2σ(*F* ^2^)] = 0.045
*wR*(*F* ^2^) = 0.109
*S* = 1.02
2092 reflections
285 parameters
6 restraints
H atoms treated by a mixture of independent and constrained refinement
Δρ_max_ = 0.18 e Å^−3^
Δρ_min_ = −0.22 e Å^−3^

### 

Data collection: *SMART* (Bruker, 1998 ▶); cell refinement: *SAINT* (Bruker, 1998 ▶); data reduction: *SAINT*; program(s) used to solve structure: *SHELXTL* (Sheldrick, 2008 ▶); program(s) used to refine structure: *SHELXTL*; molecular graphics: *SHELXTL*; software used to prepare material for publication: *SHELXTL* and *PLATON* (Spek, 2009 ▶).

## Supplementary Material

Crystal structure: contains datablocks global, I. DOI: 10.1107/S1600536810014017/fb2190sup1.cif

Structure factors: contains datablocks I. DOI: 10.1107/S1600536810014017/fb2190Isup2.hkl

Additional supplementary materials:  crystallographic information; 3D view; checkCIF report

## Figures and Tables

**Table 1:** Hydrogen-bond geometry (Å, °)

*D*—H⋯*A*	*D*—H	H⋯*A*	*D*⋯*A*	*D*—H⋯*A*
N3—H3*C*⋯N1^i^	0.89	2.15	3.032 (4)	170
N3—H3*B*⋯O7^ii^	0.89	1.90	2.787 (4)	171
N3—H3*A*⋯O3	0.89	2.01	2.894 (4)	170
N2—H2*A*⋯O5^iii^	0.90 (1)	2.06 (2)	2.922 (4)	161 (4)
O7—H7⋯O5^iii^	0.86 (1)	1.96 (3)	2.762 (4)	155 (5)
O1—H1⋯O6^iii^	0.90 (5)	1.58 (6)	2.479 (3)	177 (5)
O4—H4⋯O2^iv^	0.88 (1)	1.79 (2)	2.611 (3)	155 (5)
C20—H20*A*⋯O6^v^	0.97	2.59	3.200 (6)	122

Symmetry codes: (i) 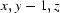; (ii) 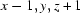; (iii) 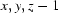; (iv) 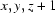; (v) 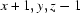.

**Table 2:** π–π inter­actions (Å) *Cg*1, *Cg*2 and *Cg*3 are the centroids of the N2,C9,C8,C14,C15 (pyrrole), C8–C13 (benzene) and N1,C1–C5 (pyridine) rings, respectively.

*Cg*1⋯*Cg*3^i^	3.665 (2)	*Cg*2⋯*Cg*3^i^	3.722 (2)
*Cg*1⋯*Cg*3^ii^	3.683 (2)	*Cg*2⋯*Cg*3^ii^	3.701 (2)

Symmetry codes: (i) 1 + *x*, −1 + *y*, *z*; (ii) *x*, −1 + *y*, *z*.

**Fig. 1.**
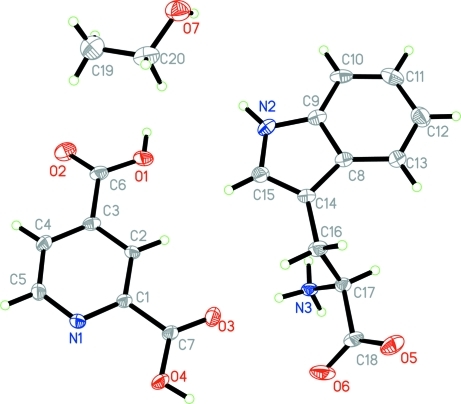
The constituing molecules of the title structure, showing 30% probability displacement ellipsoids and the atom-numbering scheme.

**Fig. 2.**
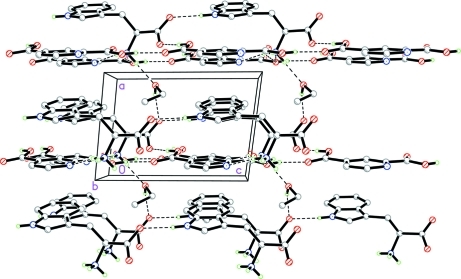
Molecular packing of the compound, viewed along the b axis. The hydrogen bonds are shown as dashed lines.

## supplementary crystallographic information

### Comment

Supramolecular assemblies are interesting field in the design of new and complicated materials. The compounds bearing imino, carboxylate and pyridine groups readily form supramolecular structures via intermolecular hydrogen bonds (Pourayoubi et al., 2010; Broker & Tiekink, 2010; Hemamalini & Fun, 2010; Narimani & Yamin, 2010). The present paper reports a new supramolecular structure of the title compound.

The title compound consists of (2S*)-2-amino-3-(1H-indol-3-yl)propionic acid molecule in the zwitterionic form, pyridine-2,4-dicarboxylic acid molecule and ethanol molecule (Fig. 1). In the crystal structure, (2S^*^)-2-amino-3-(1H-indol-3-yl)propionic acid molecules and pyridine-2,4-dicarboxylic acid molecules are linked via the strong intermolecular O—H···O and intermolecular N—H···O hydrogen bonds (Desiraju & Steiner, 1999), see Tab. 1. These molecules form layers parallel to the plane (1 0 0). The layers are further linked with ethanol molecules via weaker intermolecular O–H···O hydrogen bonds (Tab. 1) forming the three-dimensional network (Fig. 2). There is also a C–H···O weak hydrogen bond (Tab. 1). Moreover, there are also π-electron ring—π-electron ring interactions in the structure that are specified in Tab. 2 (Spek, 2009).

The difference electron density map contained some peaks in the vicinity of O5 and O6 along the direction O1-H1 and O7-H7. However, the C18-O5 (1.235 (4) Å) and C18-O6 (1.242 (4) Å) distances corresponded well to the unprotonated C-O distances in the carboxyl group. The search in the Cambridge Crystallographic Database (version 5.31 with addenda up to February 26, 2010; Allen, 2002) has revealed that the average C-O distance in the carboxyl is 1.251 (1) Å from 1269 observations, <i. e.> close to the distances C18-O5 and C18-O6.

### Experimental

Equimolar quantities (1.0 mmol each) of (2*S**)-2-amino-3-(1*H*-indol-3-yl)propionic acid (L-tryptophan) (204 mg) and pyridine-2,4-dicarboxylic acid (167 mg) were mixed in solution (50 ml) of ethanol and water (v:v = 1:1). The mixture was stirred at room temperature for 3 h to give a colourless solution. After keeping the solution in air for 15 d, colourless block-shaped crystals with average size of 0.3 mm × 0.2 mm × 0.2 mm developed.

### Refinement

All the H atoms have been observed in the difference electron density maps. The atoms attached to C atoms were have been constrained: C_aryl_-H, C_methylene_-H, C_methyl_-H = 0.93, 0.97, 0.96 Å, respectively. U_iso_(H_aryl_)=1.2U_eq_(C_aryl_), U_iso_(H_methylene_)=1.2U_eq_(C_methylene_), U_iso_(H_methyl_)=1.5U_eq_(C_methyl_). The hydrogens from N3 have also been constrained: N_ammonium_-H = 0.89 Å; U_iso_(H)=1.5U_eq_(N3). The hydrogens involved in the strongest hydrogen bonds (Tab. 1) have been treated differently: The positional parameters of H1 have been refined freely; those of H4 and H7 with the distance restraints 0.88 (1) and 0.85 (1) Å, respectively, H2A with the distance restraint 0.90 (1) Å. The values for these distance restraints have been retrieved from the Cambridge Crystallographic Database (version 5.31 with addenda up to February 26, 2010; Allen, 2002) on the reliably determined structures. The displacement parameters *U*_iso_ of H1, H4, H7 and H2A equaled 1.5×*U*_eq_ of the respective carrier atoms. In the absence of significant anomalous scattering effects 1628 Friedel pairs have been merged. The absolute structure has been determined from known configuration of (2*S**)-2-amino-3-(1*H*-indol-3-yl)propionic acid (L-tryptophan) used in the preparation.

### Crystal data

**Table:**

C_11_H_12_N_2_O_2_·C_7_H_5_NO_4_·C_2_H_6_O	*Z* = 1
*M**_r_* = 417.41	*F*(000) = 220
Triclinic, *P*1	*D*_x_ = 1.411 Mg m^−^^3^
Hall symbol: P 1	Mo *K*α radiation, λ = 0.71073 Å
*a* = 7.0320 (14) Å	Cell parameters from 1200 reflections
*b* = 7.7590 (16) Å	θ = 2.7–24.0°
*c* = 9.5800 (19) Å	µ = 0.11 mm^−^^1^
α = 85.44 (3)°	*T* = 298 K
β = 81.89 (3)°	Block, colourless
γ = 71.84 (3)°	0.27 × 0.23 × 0.22 mm
*V* = 491.34 (19) Å^3^	

### Data collection

**Table:**

Bruker SMART 1000 CCD diffractometer	2092 independent reflections
Radiation source: fine-focus sealed tube	1815 reflections with *I* > 2σ(*I*)
graphite	*R*_int_ = 0.024
ω scans	θ_max_ = 27.0°, θ_min_ = 2.2°
Absorption correction: multi-scan (*SADABS*; Sheldrick, 1996)	*h* = −8→8
*T*_min_ = 0.971, *T*_max_ = 0.977	*k* = −9→9
4115 measured reflections	*l* = −12→12

### Refinement

**Table:**

Refinement on *F*^2^	Primary atom site location: structure-invariant direct methods
Least-squares matrix: full	Secondary atom site location: difference Fourier map
*R*[*F*^2^ > 2σ(*F*^2^)] = 0.045	Hydrogen site location: difference Fourier map
*wR*(*F*^2^) = 0.109	H atoms treated by a mixture of independent and constrained refinement
*S* = 1.02	*w* = 1/[σ^2^(*F*_o_^2^) + (0.0628*P*)^2^] where *P* = (*F*_o_^2^ + 2*F*_c_^2^)/3
2092 reflections	(Δ/σ)_max_ < 0.001
285 parameters	Δρ_max_ = 0.18 e Å^−^^3^
6 restraints	Δρ_min_ = −0.21 e Å^−^^3^
78 constraints	

### Special details

**Table:**

Geometry. All esds (except the esd in the dihedral angle between two l.s. planes) are estimated using the full covariance matrix. The cell esds are taken into account individually in the estimation of esds in distances, angles and torsion angles; correlations between esds in cell parameters are only used when they are defined by crystal symmetry. An approximate (isotropic) treatment of cell esds is used for estimating esds involving l.s. planes.
Refinement. Refinement of F^2^ against ALL reflections. The weighted R-factor wR and goodness of fit S are based on F^2^, conventional R-factors R are based on F, with F set to zero for negative F^2^. The threshold expression of F^2^ > 2sigma(F^2^) is used only for calculating R-factors(gt) etc. and is not relevant to the choice of reflections for refinement. R-factors based on F^2^ are statistically about twice as large as those based on F, and R- factors based on ALL data will be even larger.

### Fractional atomic coordinates and isotropic or equivalent isotropic displacement parameters (Å^2^)

**Table:**

	*x*	*y*	*z*	*U*_iso_*/*U*_eq_	
O1	0.2726 (4)	0.7005 (3)	0.5137 (3)	0.0503 (7)	
H1	0.276 (7)	0.635 (7)	0.439 (6)	0.075*	
O2	0.1776 (6)	0.9447 (4)	0.3743 (3)	0.0725 (10)	
O3	0.2236 (5)	0.7586 (4)	1.0219 (3)	0.0560 (8)	
O4	0.1597 (5)	1.0309 (3)	1.1063 (2)	0.0509 (7)	
H4	0.175 (7)	0.970 (6)	1.187 (3)	0.076*	
O5	0.5693 (4)	0.2870 (4)	1.3453 (3)	0.0522 (7)	
O6	0.2941 (4)	0.5149 (4)	1.3097 (3)	0.0554 (8)	
O7	0.9134 (4)	0.3938 (4)	0.3005 (3)	0.0601 (8)	
H7	0.831 (6)	0.332 (6)	0.301 (6)	0.090*	
N1	0.1492 (4)	1.1892 (3)	0.8482 (3)	0.0316 (6)	
N2	0.5947 (4)	0.2711 (4)	0.6481 (3)	0.0396 (7)	
H2A	0.582 (7)	0.304 (6)	0.5569 (17)	0.059*	
N3	0.2260 (4)	0.3869 (3)	1.0847 (3)	0.0304 (6)	
H3A	0.2096	0.5039	1.0629	0.046*	
H3B	0.1305	0.3763	1.1534	0.046*	
H3C	0.2174	0.3316	1.0090	0.046*	
C1	0.1801 (5)	1.0105 (4)	0.8599 (3)	0.0274 (6)	
C2	0.2031 (5)	0.9032 (4)	0.7466 (3)	0.0288 (7)	
H2	0.2292	0.7783	0.7597	0.035*	
C3	0.1866 (5)	0.9845 (4)	0.6140 (3)	0.0302 (7)	
C4	0.1478 (5)	1.1699 (5)	0.6004 (3)	0.0362 (8)	
H4A	0.1329	1.2299	0.5128	0.043*	
C5	0.1316 (5)	1.2645 (4)	0.7195 (3)	0.0346 (7)	
H5	0.1068	1.3895	0.7090	0.042*	
C6	0.2122 (5)	0.8722 (5)	0.4871 (3)	0.0400 (8)	
C7	0.1919 (5)	0.9201 (4)	1.0033 (3)	0.0329 (7)	
C8	0.6594 (5)	0.0930 (5)	0.8419 (3)	0.0311 (7)	
C9	0.6549 (5)	0.0934 (5)	0.6954 (3)	0.0341 (7)	
C10	0.7065 (6)	−0.0663 (5)	0.6231 (4)	0.0451 (9)	
H10	0.7036	−0.0642	0.5263	0.054*	
C11	0.7617 (6)	−0.2262 (5)	0.6995 (5)	0.0502 (10)	
H11	0.7968	−0.3349	0.6535	0.060*	
C12	0.7666 (6)	−0.2308 (6)	0.8447 (5)	0.0510 (10)	
H12	0.8051	−0.3420	0.8938	0.061*	
C13	0.7152 (5)	−0.0727 (5)	0.9160 (4)	0.0402 (8)	
H13	0.7178	−0.0767	1.0130	0.048*	
C14	0.6009 (5)	0.2784 (4)	0.8802 (3)	0.0314 (7)	
C15	0.5622 (5)	0.3797 (5)	0.7600 (4)	0.0354 (7)	
H15	0.5194	0.5059	0.7544	0.043*	
C16	0.5926 (5)	0.3425 (5)	1.0252 (3)	0.0368 (8)	
H16A	0.5705	0.4726	1.0191	0.044*	
H16B	0.7223	0.2856	1.0590	0.044*	
C17	0.4276 (5)	0.3016 (4)	1.1330 (3)	0.0303 (7)	
H17	0.4531	0.1699	1.1423	0.036*	
C18	0.4303 (5)	0.3726 (5)	1.2774 (3)	0.0349 (7)	
C19	0.7449 (10)	0.7082 (7)	0.2595 (6)	0.0880 (18)	
H19A	0.6650	0.6699	0.2022	0.132*	
H19B	0.8566	0.7329	0.2008	0.132*	
H19C	0.6635	0.8162	0.3066	0.132*	
C20	0.8210 (7)	0.5635 (6)	0.3650 (5)	0.0608 (11)	
H20A	0.9183	0.5939	0.4129	0.073*	
H20B	0.7101	0.5543	0.4350	0.073*	

### Atomic displacement parameters (Å^2^)

**Table:**

	*U*^11^	*U*^22^	*U*^33^	*U*^12^	*U*^13^	*U*^23^
O1	0.0771 (19)	0.0433 (16)	0.0285 (13)	−0.0102 (14)	−0.0109 (12)	−0.0145 (11)
O2	0.128 (3)	0.066 (2)	0.0212 (13)	−0.0198 (19)	−0.0207 (15)	−0.0064 (13)
O3	0.105 (2)	0.0348 (14)	0.0324 (14)	−0.0252 (15)	−0.0166 (14)	0.0061 (11)
O4	0.100 (2)	0.0418 (15)	0.0188 (11)	−0.0313 (15)	−0.0099 (13)	0.0001 (10)
O5	0.0589 (17)	0.0725 (19)	0.0293 (13)	−0.0211 (14)	−0.0158 (12)	−0.0036 (13)
O6	0.0627 (18)	0.0611 (18)	0.0423 (16)	−0.0106 (14)	−0.0094 (13)	−0.0298 (13)
O7	0.0569 (18)	0.0624 (19)	0.0623 (19)	−0.0243 (14)	0.0092 (15)	−0.0139 (15)
N1	0.0417 (15)	0.0273 (14)	0.0255 (13)	−0.0086 (11)	−0.0053 (11)	−0.0044 (11)
N2	0.0477 (17)	0.0486 (18)	0.0227 (14)	−0.0155 (14)	−0.0048 (12)	0.0021 (13)
N3	0.0396 (15)	0.0306 (14)	0.0244 (13)	−0.0147 (12)	−0.0020 (10)	−0.0080 (10)
C1	0.0315 (15)	0.0293 (15)	0.0226 (14)	−0.0100 (12)	−0.0040 (11)	−0.0028 (12)
C2	0.0369 (16)	0.0255 (15)	0.0235 (15)	−0.0069 (13)	−0.0046 (12)	−0.0063 (12)
C3	0.0294 (16)	0.0399 (18)	0.0219 (14)	−0.0090 (14)	−0.0053 (12)	−0.0062 (13)
C4	0.047 (2)	0.0393 (19)	0.0226 (16)	−0.0142 (16)	−0.0075 (14)	0.0034 (14)
C5	0.0436 (18)	0.0273 (16)	0.0329 (17)	−0.0100 (14)	−0.0083 (14)	0.0030 (13)
C6	0.050 (2)	0.046 (2)	0.0230 (18)	−0.0117 (17)	−0.0045 (15)	−0.0066 (15)
C7	0.0490 (19)	0.0331 (19)	0.0219 (15)	−0.0177 (15)	−0.0084 (13)	−0.0028 (13)
C8	0.0297 (16)	0.0371 (17)	0.0276 (15)	−0.0126 (13)	0.0003 (12)	−0.0050 (13)
C9	0.0324 (17)	0.0437 (19)	0.0283 (16)	−0.0144 (15)	−0.0017 (13)	−0.0053 (15)
C10	0.051 (2)	0.056 (2)	0.0306 (18)	−0.0194 (18)	0.0049 (15)	−0.0206 (17)
C11	0.046 (2)	0.044 (2)	0.061 (3)	−0.0128 (18)	0.0051 (19)	−0.022 (2)
C12	0.051 (2)	0.038 (2)	0.059 (3)	−0.0112 (17)	0.0027 (19)	−0.0013 (18)
C13	0.043 (2)	0.040 (2)	0.0339 (18)	−0.0092 (16)	−0.0002 (14)	0.0002 (16)
C14	0.0327 (17)	0.0356 (17)	0.0255 (15)	−0.0096 (14)	0.0001 (12)	−0.0075 (13)
C15	0.0391 (18)	0.0355 (18)	0.0319 (17)	−0.0134 (15)	0.0004 (14)	−0.0025 (14)
C16	0.0455 (19)	0.0410 (19)	0.0288 (17)	−0.0209 (15)	0.0004 (14)	−0.0071 (14)
C17	0.0397 (17)	0.0310 (16)	0.0241 (15)	−0.0147 (13)	−0.0050 (12)	−0.0051 (12)
C18	0.0426 (19)	0.046 (2)	0.0233 (15)	−0.0229 (16)	−0.0025 (13)	−0.0059 (14)
C19	0.122 (5)	0.070 (4)	0.056 (3)	−0.013 (3)	−0.001 (3)	0.002 (3)
C20	0.061 (3)	0.074 (3)	0.046 (2)	−0.023 (2)	0.0042 (19)	−0.013 (2)

### Geometric parameters (Å, °)

**Table:**

O1—C6	1.282 (5)	C5—H5	0.9300
O1—H1	0.90 (5)	C8—C13	1.390 (5)
O2—C6	1.199 (4)	C8—C9	1.408 (5)
O3—C7	1.205 (4)	C8—C14	1.430 (5)
O4—C7	1.310 (4)	C9—C10	1.389 (5)
O4—H4	0.875 (11)	C10—C11	1.364 (6)
O5—C18	1.235 (4)	C10—H10	0.9300
O6—C18	1.242 (4)	C11—C12	1.393 (6)
O7—C20	1.422 (5)	C11—H11	0.9300
O7—H7	0.855 (11)	C12—C13	1.374 (5)
N1—C5	1.326 (4)	C12—H12	0.9300
N1—C1	1.333 (4)	C13—H13	0.9300
N2—C15	1.367 (4)	C14—C15	1.352 (5)
N2—C9	1.370 (5)	C14—C16	1.500 (4)
N2—H2A	0.899 (11)	C15—H15	0.9300
N3—C17	1.488 (4)	C16—C17	1.534 (4)
N3—H3A	0.8900	C16—H16A	0.9700
N3—H3B	0.8900	C16—H16B	0.9700
N3—H3C	0.8900	C17—C18	1.534 (4)
C1—C2	1.381 (4)	C17—H17	0.9800
C1—C7	1.493 (4)	C19—C20	1.472 (7)
C2—C3	1.376 (4)	C19—H19A	0.9600
C2—H2	0.9300	C19—H19B	0.9600
C3—C4	1.377 (5)	C19—H19C	0.9600
C3—C6	1.509 (4)	C20—H20A	0.9700
C4—C5	1.378 (5)	C20—H20B	0.9700
C4—H4A	0.9300		
			
C6—O1—H1	113 (3)	C10—C11—C12	121.7 (4)
C7—O4—H4	110 (3)	C10—C11—H11	119.2
C20—O7—H7	112 (4)	C12—C11—H11	119.2
C5—N1—C1	116.2 (3)	C13—C12—C11	120.7 (4)
C15—N2—C9	108.7 (3)	C13—C12—H12	119.6
C15—N2—H2A	128 (3)	C11—C12—H12	119.6
C9—N2—H2A	123 (3)	C12—C13—C8	119.4 (3)
C17—N3—H3A	109.5	C12—C13—H13	120.3
C17—N3—H3B	109.5	C8—C13—H13	120.3
H3A—N3—H3B	109.5	C15—C14—C8	106.3 (3)
C17—N3—H3C	109.5	C15—C14—C16	128.1 (3)
H3A—N3—H3C	109.5	C8—C14—C16	125.6 (3)
H3B—N3—H3C	109.5	C14—C15—N2	110.7 (3)
N1—C1—C2	123.8 (3)	C14—C15—H15	124.7
N1—C1—C7	118.4 (3)	N2—C15—H15	124.7
C2—C1—C7	117.8 (3)	C14—C16—C17	114.1 (3)
C3—C2—C1	118.8 (3)	C14—C16—H16A	108.7
C3—C2—H2	120.6	C17—C16—H16A	108.7
C1—C2—H2	120.6	C14—C16—H16B	108.7
C2—C3—C4	118.3 (3)	C17—C16—H16B	108.7
C2—C3—C6	120.5 (3)	H16A—C16—H16B	107.6
C4—C3—C6	121.2 (3)	N3—C17—C18	109.6 (3)
C3—C4—C5	118.5 (3)	N3—C17—C16	110.3 (3)
C3—C4—H4A	120.7	C18—C17—C16	110.3 (3)
C5—C4—H4A	120.7	N3—C17—H17	108.8
N1—C5—C4	124.3 (3)	C18—C17—H17	108.8
N1—C5—H5	117.8	C16—C17—H17	108.8
C4—C5—H5	117.8	O5—C18—O6	127.7 (3)
O2—C6—O1	125.9 (3)	O5—C18—C17	117.1 (3)
O2—C6—C3	120.3 (3)	O6—C18—C17	115.1 (3)
O1—C6—C3	113.8 (3)	C20—C19—H19A	109.5
O3—C7—O4	123.2 (3)	C20—C19—H19B	109.5
O3—C7—C1	122.3 (3)	H19A—C19—H19B	109.5
O4—C7—C1	114.5 (3)	C20—C19—H19C	109.5
C13—C8—C9	118.6 (3)	H19A—C19—H19C	109.5
C13—C8—C14	134.3 (3)	H19B—C19—H19C	109.5
C9—C8—C14	107.1 (3)	O7—C20—C19	111.0 (4)
N2—C9—C10	130.7 (3)	O7—C20—H20A	109.4
N2—C9—C8	107.2 (3)	C19—C20—H20A	109.4
C10—C9—C8	122.1 (3)	O7—C20—H20B	109.4
C11—C10—C9	117.6 (3)	C19—C20—H20B	109.4
C11—C10—H10	121.2	H20A—C20—H20B	108.0
C9—C10—H10	121.2		

### Hydrogen-bond geometry (Å, °)

**Table:**

*D*—H···*A*	*D*—H	H···*A*	*D*···*A*	*D*—H···*A*
N3—H3C···N1^i^	0.89	2.15	3.032 (4)	170
N3—H3B···O7^ii^	0.89	1.90	2.787 (4)	171
N3—H3A···O3	0.89	2.01	2.894 (4)	170
N2—H2A···O5^iii^	0.90 (1)	2.06 (2)	2.922 (4)	161 (4)
O7—H7···O5^iii^	0.86 (1)	1.96 (3)	2.762 (4)	155 (5)
O1—H1···O6^iii^	0.90 (5)	1.58 (6)	2.479 (3)	177 (5)
O4—H4···O2^iv^	0.88 (1)	1.79 (2)	2.611 (3)	155 (5)
C20—H20A···O6^v^	0.97	2.59	3.200 (6)	122

Symmetry codes: (i) *x*, *y*−1, *z*; (ii) *x*−1, *y*, *z*+1;  (iii) *x*, *y*, *z*−1; (iv) *x*, *y*, *z*+1; (v) *x*+1, *y*, *z*−1.

### Table 2 Overview of π-electron—π-electron ring interactions (Å) in the structure

Cg1, Cg2 and Cg3 are the centroids of the N2,C9,C8,C14,C15 (pyrrole), C8–C13 (benzene) and N1,C1–C5 (pyridine), respectively.

**Table:**

Cg···Cg	distance	Cg···Cg	distance
Cg1···Cg3^i^	3.665 (2)	Cg2···Cg3^i^	3.722 (2)
Cg1···Cg3^ii^	3.683 (2)	Cg2···Cg3^ii^	3.701 (2)

Symmetry codes: (i) 1+*x*, -1+*y*, *z*; (ii) *x*, -1+*y*, *z*.
